# Ultrasound-guided bilateral combined inguinal femoral and subgluteal sciatic nerve blocks for simultaneous bilateral below-knee amputations due to bilateral diabetic foot gangrene unresponsive to peripheral arterial angioplasty and bypass surgery in a coagulopathic patient on antiplatelet therapy with a history of percutaneous coronary intervention for ischemic heart disease: A case report

**DOI:** 10.1097/MD.0000000000004324

**Published:** 2016-07-22

**Authors:** Sung Hye Byun, Jonghoon Lee, Jong Hae Kim

**Affiliations:** Department of Anesthesiology and Pain Medicine, School of Medicine, Catholic University of Daegu, Daegu, Republic of Korea.

**Keywords:** amputation, coronary artery disease, femoral nerve, nerve block, sciatic nerve

## Abstract

**Background:**

Patients on antiplatelet therapy following percutaneous coronary intervention can become coagulopathic due to infection. Performing regional anesthesia for bilateral surgery in such cases is challenging. We report a case of successful combined inguinal femoral and subgluteal sciatic nerve blocks (CFSNBs) for simultaneous bilateral below-knee amputations in a coagulopathic patient on antiplatelet therapy.

**Methods:**

A 70-year-old male patient presented with pain in both feet due to diabetic foot syndrome. The condition could not be managed by open amputations of the toes at the metatarsal bones and subsequent antibiotic therapy. Computed tomographic angiography showed significant stenosis in the arteries supplying the lower limbs, indicating atherosclerotic gangrene in both feet. Balloon angioplasty and bypass surgery with subsequent debridements with application of negative-pressure wound therapy and additional open amputations did not improve the patient's clinical condition: his leukocyte counts and C-reactive protein levels were above the normal range, and his prothrombin and activated partial thromboplastin times were increased.

**Results:**

Simultaneous bilateral below-knee amputations were performed under ultrasound-guided CFSNBs. Following left CFSNBs using 45 mL of a local anesthetic mixture (1:1 ratio of 1.0% mepivacaine and 0.75% ropivacaine), the left below-knee amputation was performed for 76 minutes. Subsequently, under right CFSNBs using 47 mL of the local anesthetic mixture, the right below-knee amputation proceeded for 85 minutes. Throughout each surgery, dexmedetomidine was continuously administered, and a sensory blockade was well maintained in both limbs. The patient did not complain of pain due to regression of the first CFSNBs during the second surgery. The CFSNBs successfully prevented tourniquet pain. Local anesthetic systemic toxicity (LAST) and hemodynamic instability due to tourniquet deflation and administration of dexmedetomidine did not occur. No additional analgesic was required to supplement insufficient surgical anesthesia. Postoperatively, no neurologic complications related to the CFSNBs were reported.

**Conclusion:**

The timely placement of bilateral CFSNBs immediately before the corresponding limb surgery, which lasted for less than 2 hours, provided successful surgical anesthesia in both lower limbs without LAST or pain due to regression of the CFSNBs that were performed during the first surgery.

## 1 Introduction

The placement of peripheral nerve blocks for bilateral limb surgery is challenging because increases in the required dose of a local anesthetic lead to increases in the risk of local anesthetic systemic toxicity (LAST) and other side effects related to regional anesthesia. Furthermore, this procedure prolongs anesthesia preparation time, leading to a delay in surgery. To date, several reports have been published on the use of bilateral brachial plexus blocks for surgical anesthesia^[1–3]^ or postoperative pain management along with general anesthesia.^[4–6]^ Bilateral blocks are also performed for nonsurgical reasons.^[7]^ Theoretically, due to its relatively compact topographical arrangement, the brachial plexus could be easily blocked by a single local anesthetic injection that provides anesthesia to nearly the entire area of the upper limb. In contrast, the entirety of the lumbosacral plexus that innervates a lower limb cannot be anesthetized with a single injection. Therefore, the lumbar and sacral plexuses should be separately blocked for complete unilateral lower limb anesthesia, necessitating a larger dose of local anesthetics than that used for brachial plexus blocks. If the blocks are performed bilaterally, LAST cannot be easily averted. For this reason, neuraxial or general anesthesia has been preferred to bilateral peripheral nerve blocks. However, neuraxial or general anesthesia is frequently contraindicated in patients undergoing major lower extremity amputation due to the need for concomitant administration of anticoagulants and/or antiplatelet agents or because of comorbidities that cause anesthesia-related hemodynamic instability. In addition, major lower extremity amputation for peripheral arterial disease that is complicated by gangrene carries a 2-year mortality rate of approximately 30%.^[8]^ Herein, we report our success in performing combined inguinal femoral and subgluteal sciatic nerve blocks (CFSNBs) immediately before corresponding limb amputations (to reduce the risk of LAST) for bilateral diabetic gangrene in a patient on antiplatelet therapy who became coagulopathic due to infection. The gangrene was unresponsive to a previously performed peripheral arterial angiography and bypass surgery.

## 2 Case report

A 70-year-old male patient (167 cm, 62 kg) presented with pain in both feet due to diabetes mellitus. He underwent open amputations of the right 3rd to 5th and left 4th and 5th metatarsal bones in a local medical clinic. However, due to refractory abscesses around the operation sites that were unresponsive to antibiotic therapy, the patient was transferred to our hospital 13 days after the operations for further evaluation and treatment. On admission, he was given 60 mg of gliclazide and 1000 mg of metformin per day for diabetes mellitus that had been diagnosed 40 years previously. He received 50 mg of atenolol, 12.5 mg of chlorthalidone, 4 mg of benidipine, and 100 mg of aspirin per day for hypertension that had been diagnosed 30 years previously and was complicated with chronic ischemic heart disease, as demonstrated by total occlusion of the left anterior descending artery on coronary angiography. The occlusion was treated with a percutaneous coronary intervention 3 years previously. The above medications were maintained during the admission period. A complete blood cell count revealed a leukocyte count of 14,800 cells/μL, a hemoglobin level of 9.8 g/dL, and a platelet count of 452,000 cells/μL. Liver function tests, prothrombin time, activated partial thromboplastin time, serum electrolytes, sodium levels, and creatinine levels were within normal ranges. Transthoracic echocardiography indicated apical septal hypokinesia, left ventricular concentric hypertrophy, mild diastolic dysfunction of the left ventricle, decreased mobility of the noncoronary and left coronary cusps of the aortic valve, and a left ventricular ejection fraction of 61%. Computed tomographic angiography showed significant stenosis in the bilateral tibial, dorsalis pedis, and plantar arteries and diffuse atherosclerosis with calcified plaques in the aorta and pelvic and femoral arteries, indicating atherosclerotic gangrene in both feet. Daily administration of alprostadil (10 μg/d) was started to dilate the stenotic vessels and maintain their blood flow and continued until the bilateral below-knee amputations were performed. Under epidural anesthesia, balloon angioplasties in the left posterior tibial and peroneal arteries and a right above-knee popliteal artery to right posterior tibial artery bypass with a reversed left great saphenous vein were respectively performed 2 and 7 days after the admission. Despite subsequent debridements with application of negative pressure and additional open amputations of the right 2nd and 3rd metatarsal and 1st proximal phalangeal bones and the left 2nd metatarsal bone and 1 metatarsophalangeal joint under spinal anesthesia, the status of the wound did not improve. Therefore, simultaneous bilateral below-knee amputations were planned 49 days after the admission. The leukocyte count and C-reactive protein level were maintained above normal ranges (11,000–14,000 cells/μL and more than 30 mg/L, respectively) during admission, and the prothrombin time and activated partial thromboplastin time were increased to 15.3 and 55.6 seconds, respectively, from 1 month before the bilateral below-knee amputations.

**Figure 1:**
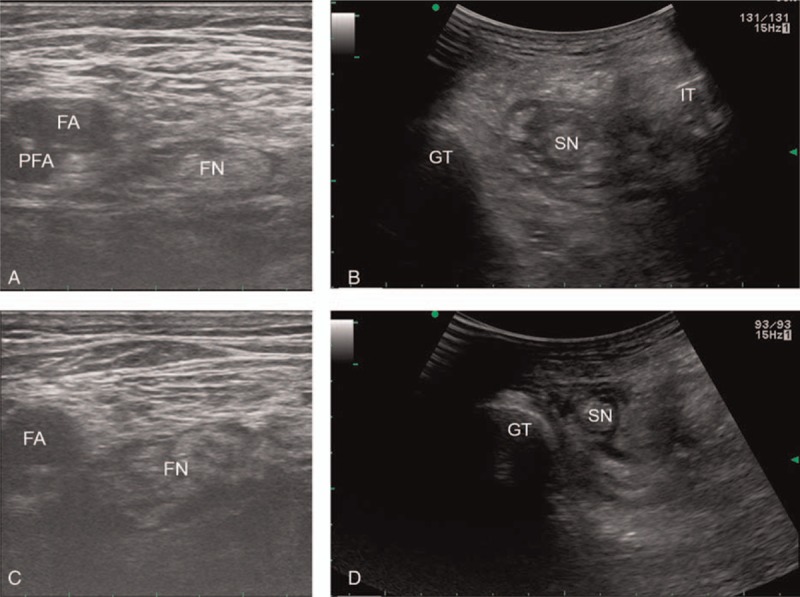
Bilateral combined inguinal femoral and subgluteal sciatic nerve blocks. The bilateral femoral and sciatic nerves were appropriately surrounded by local anesthetics. (A) Left femoral nerve block. (B) Left sciatic nerve block. (C) Right femoral nerve block. (D) Right sciatic nerve block. FA = femoral artery, FN = femoral nerve, GT = greater trochanter, IT = ischial tuberosity, PFA = profunda femoris artery, SN = sciatic nerve.

**Figure 2:**
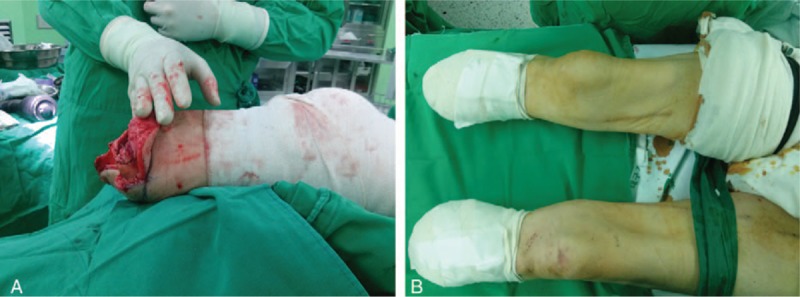
Bilateral below-knee amputations. (A) Right leg amputated below the knee during the surgery. (B) Both legs amputated below the knee at the end of the surgery.

Upon arrival in the operating room, electrocardiography, pulse oximetry, and noninvasive blood pressure monitoring were instituted. After draping the left inguinal region, the femoral nerve was identified lateral to the femoral artery using a 5- to 13-MHz linear phased array transducer (UST-5411; Hitachi Aloka Medical, Ltd., Tokyo, Japan) equipped on an ultrasound machine (ProSound α7 Premier; Hitachi Aloka Medical). Under ultrasound guidance, a 23-ga Tuohy needle was introduced toward the femoral nerve parallel to the ultrasound beam, and 21 mL of a local anesthetic mixture containing 30 mL of 1.0% mepivacaine and 30 mL of 0.75% ropivacaine was injected through the needle near the femoral nerve (Fig. 1A). The trajectory of the needle was adjusted to achieve even distribution of the local anesthetics around the femoral nerve. Then, the patient was placed in the right lateral position with the left hip and knee joints flexed by 30° to 50°. Following the identification of the left sciatic nerve located in the intermuscular plane of the gluteus maximus and medius muscles between the ipsilateral ischial tuberosity and greater trochanter using a convex phased array transducer (UST-9130, Hitachi Aloka Medical, Ltd.), 24 mL of the local anesthetic mixture was placed near the sciatic nerve through the 23-ga Tuohy needle. The needle was introduced from the lateral side using the in-plane technique (Fig. 1B). While waiting for the complete effect of the CFSNBs, the left brachial artery was catheterized under ultrasound guidance for continuous arterial blood pressure monitoring and arterial blood gas analysis after a failed attempt to catheterize the left radial artery, in which blood flow was not recognized by color Doppler spectrometry. Continuous intravenous administration of dexmedetomidine (0.3 μg/kg/h) was initiated following a bolus administration (0.5 μg/kg). After confirmation of the sensory blockade using a pinch test on the dermatomes corresponding to the femoral and sciatic nerves (23 minutes after the end of local anesthetic injection for each block), the surgery for the left below-knee amputation began following blood exsanguination with an Esmarch bandage from the whole left lower extremity, with subsequent inflation of a tourniquet cuffed around the thigh. After the end of the surgery, which lasted for 76 minutes, CFSNBs were performed in the right lower limb using 17 and 30 mL of the local anesthetic mixture for femoral nerve and sciatic nerve blocks, respectively (Fig. 1C and D). Following the inflation of the tourniquet, the surgery for the right limb amputation began after the sensory blockade of the lower limb was confirmed (Fig. 2A). Throughout the bilateral limb amputations (Fig. 2B), the sensory blockades were well maintained in both limbs. The patient did not complain of pain due to the regression of the first CFSNBs during the second surgery. The CFSNBs also successfully managed pain caused by tourniquet inflation. No hemodynamic instability, such as hypotension and/or bradycardia, occurred in response to CFSNBs-associated sympathectomy, tourniquet deflation, or dexmedetomidine administration. LAST did not occur. No additional analgesic was required to supplement incomplete surgical anesthesia. The procedural time for each CFSNB was 2 minutes for the bilateral femoral nerve blocks, 5 minutes for the left sciatic nerve block, and 8 minutes for the right sciatic nerve block. Postoperatively, no neurologic complications related to the CFSNBs and no hemodynamic instability were reported. The patient was discharged without any significant events 25 days after the surgery.

The approval from the institutional review board was not required because the clinical data were presented anonymously and the written informed consent was obtained from the patient.

## 3 Discussion

A recent retrospective review of 407 patients undergoing major lower extremity amputation showed comparable operative mortality and incidence of postoperative myocardial infarction between patients receiving a neuraxial block (spinal and epidural anesthesia) and patients receiving general anesthesia.^[9]^ However, a reduced incidence of postoperative pulmonary complications and cardiac arrhythmias, a decreased duration of hospital and intensive care unit stays, and a reduced need for transfusion made the neuraxial block more favorable for major lower extremity amputation than general anesthesia.^[9]^ In the present case, our patient had a history of myocardial infarction and hypertension, which are risk factors for cardiovascular death within 30 days after elective surgery under general anesthesia.^[10]^ Therefore, a neuraxial block appeared to be more beneficial for our patient than general anesthesia. However, despite its advantages over general anesthesia, a neuraxial block was contraindicated by the coagulopathy due to infection and ongoing antiplatelet therapy^[11]^ in this case. Furthermore, because our patient had cardiac disease, a neuraxial block might also have predisposed him to hemodynamic instability^[12,13]^, which could lead to cardiovascular events or even death.^[14]^ In contrast to general and neuraxial anesthesia, CFSNBs minimally affect hemodynamics,^[15]^ allowing for the use of dexmedetomidine, which reduces blood pressure and heart rate.^[16]^ In addition, if the procedures are performed under ultrasound guidance, blood vessel injury from a block needle would be prevented by the real-time visualization of vascular structures. Accordingly, no hemodynamic instability or hematoma formation around the needle insertion sites was observed in this case.

The total doses of 1% mepivacaine and 0.75% ropivacaine used for the 2 sessions of the CFSNBs were 460 and 345 mg, respectively. However, the total doses were administered at an interval of 115 minutes between the end of the local anesthetic injection for the 1st and 2nd CFSNBs (225 mg of 1% mepivacaine and 168.75 mg of 0.75% ropivacaine for the 1st CFSNBs and 235 mg of 1% mepivacaine and 176.25 mg of 0.75% ropivacaine for the 2nd CFSNBs). Despite the separate administration of the local anesthetics at an interval, the possibility of LAST remained. In previous studies, when 700^[17]^ and 667.2 ± 61.2 mg (mean ± standard deviation)^[18]^ of mepivacaine were administered via a single-shot technique for CFSNBs, no signs or symptoms of LAST were detected. However, an average of 667.2 mg of mepivacaine increased the maximum plasma concentration of mepivacaine (5.1 μg/mL on average)^[18]^ to the threshold needed for toxic symptoms to appear (5–6 μg/mL), whereas 700 mg of mepivacaine did not (3.91 ± 0.95 μg/mL) have the same effect.^[17]^ In addition, a threshold concentration of ropivacaine for LAST (1–2 μg/mL)^[19]^ was achieved in the absence of suggestive signs and symptoms of LAST following the administration of 300 mg of 0.75% ropivacaine mixed with 1:200,000 epinephrine for CFSNBs.^[20]^ Therefore, a dose of ropivacaine higher than that used in the above study^[20]^ and the additive effect of mepivacaine (although the dose of the mepivacaine was lower than those in the above studies^[17,18]^) would considerably increase the possibility of LAST. Moreover, the interval between the 2 block sessions (115 minutes) did not appear to help prevent the development of LAST because the local anesthetic concentration was maintained near the peak value until 90 to 120 minutes after the end of local anesthetic injection.^[17,20]^ Fortunately, our patient did not experience signs and symptoms of LAST.

Additional factors may have contributed to the prevention of LAST. For example, the use of a local anesthetic mixture has advantages over a single local anesthetic because the mixture compensates for the short durations of action of rapidly acting anesthetics (mepivacaine in this case) and the long latencies of longer acting anesthetics (ropivacaine in this case). The mixture could also decrease the incidence of cardiac events by decreasing the doses of more potent local anesthetics, the convulsion doses of which can easily cause a cardiac event.^[21]^ In addition, real-time ultrasound guidance might prevent vascular injury, which commonly leads to intravascular injection of a local anesthetic. Ultrasound imaging could also detect inadvertent intravascular entry of a block needle, thereby minimizing the amount of local anesthetic injected into a blood vessel.^[22,23]^ Furthermore, the compression of veins resulting from ultrasound transducer use reduces the risk of vascular puncture and local anesthetic injection into vessels. When a fixed dose of mepivacaine was administered for various types of nerve blocks, CFSNBs resulted in the lowest maximum plasma concentration of the anesthetic and prolonged the time to reaching the maximum plasma concentration than other types of regional anesthesia, such as lumbar epidural, caudal, intercostal-nerve (bilateral), and supraclavicular-brachial-plexus blocks.^[24]^ Finally, dexmedetomidine used for intraoperative sedation has an anticonvulsive effect by increasing the convulsive dose of local anesthetics.^[25]^ Therefore, it appears that our patient, who did not experience LAST, benefited from the above factors contributing to the prevention of LAST despite the excessive local anesthetic dose.

Even if a concern existed regarding tourniquet pain due to the omission of the obturator^[26]^ and lateral femoral cutaneous nerve blocks for the prevention of LAST, the patient did not complain of tourniquet pain during the 2 consecutive surgeries. This was presumably due to the intravenous administration of dexmedetomidine before the beginning of the surgery, as dexmedetomidine reduces sympathoadrenal responses to tourniquet pain as well as opioid requirements to relieve the pain.^[27]^

In conclusion, the timely placement of bilateral CFSNBs immediately before corresponding limb surgery that lasts for less than 2 hours provides successful surgical anesthesia in the bilateral lower limbs without resulting in LAST, hemodynamic instability, or tourniquet pain and avoids the regression of CFSNBs performed earlier during the surgery.
